# Tensin-3 Regulates Integrin-Mediated Proliferation and Differentiation of Tonsil-Derived Mesenchymal Stem Cells

**DOI:** 10.3390/cells9010089

**Published:** 2019-12-30

**Authors:** Gi Cheol Park, Hyung-Sik Kim, Hee-Young Park, Yoojin Seo, Ji Min Kim, Sung-Chan Shin, Hyun-Keun Kwon, Eui-Suk Sung, Jin-Choon Lee, Byung-Joo Lee

**Affiliations:** 1 Department of Otolaryngology, Head and Neck Surgery, Samsung Changwon Hospital, Sungkyunkwan University School of Medicine, Changwon 51353, Korea; uuhent@gmail.com; 2 Department of Life Science in Dentistry, School of Dentistry, Pusan National University, Yangsan 50612, Korea; hskimcell@pusan.ac.kr; 3 Dental and Life Science Institute, Pusan National University, Yangsan 50612, Korea; amaicat24@naver.com; 4 Department of Otorhinolaryngology, Head and Neck Surgery, Pusan National University School of Medicine and Biomedical Research Institute, Pusan National University Hospital, Busan 49241, Korea; 24hera@hanmail.net (H.-Y.P.); ny5thav@hanmail.net (J.M.K.); cha-nwi@hanmail.net (S.-C.S.); kwon-h-g@hanmail.net (H.-K.K.); 5 Department of Otorhinolaryngology, Head and Neck Surgery, Pusan National University School of Medicine and Biomedical Research Institute, Pusan National University Yangsan Hospital, Yangsan 50612, Korea; sunges77@gmail.com (E.-S.S.); ljc0209@hanmail.net (J.-C.L.)

**Keywords:** tensin-3, integrin β1, proliferation, differentiation, tonsil-derived mesenchymal stem cells

## Abstract

Human palatine tonsils are potential tissue source of multipotent mesenchymal stem cells (MSCs). The proliferation rate of palatine tonsil-derived MSCs (TMSCs) is far higher than that of bone marrow-derived MSCs (BMSCs) or adipose tissue-derived MSCs (ADSCs). In our previous study, we had found through DNA microarray analysis that tensin-3 (TNS3), a type of focal adhesion protein, was more highly expressed in TMSCs than in both BMSCs and ADSCs. Here, the role of TNS3 in TMSCs and its relationship with integrin were investigated. TNS3 expression was significantly elevated in TMSCs than in other cell types. Cell growth curves revealed a significant decrease in the proliferation and migration of TMSCs treated with siRNA for TNS3 (siTNS3). siTNS3 treatment upregulated p16 and p21 levels and downregulated SOX2 expression and focal adhesion kinase, protein kinase B, and c-Jun N-terminal kinase phosphorylation. siTNS3 transfection significantly reduced adipogenic differentiation of TMSCs and slightly decreased osteogenic and chondrogenic differentiation. Furthermore, TNS3 inhibition reduced active integrin beta-1 (ITGβ1) expression, while total ITGβ1 expression was not affected. Inhibition of ITGβ1 expression in TMSCs by siRNA showed similar results observed in TNS3 inhibition. Thus, TNS3 may play an important role in TMSC proliferation and differentiation by regulating active ITGβ1 expression.

## 1. Introduction

Recently, human palatine tonsils have emerged as a new source of mesenchymal stem cells (MSCs) [1,2,3,4]. Palatine tonsil tissue is much easier to obtain than bone marrow or adipose tissue. As tonsillectomy is performed on many children before 10 years of age, the abandoned tonsil tissues can be used for MSC banking [2]. Additionally, some palatine tonsils can be easily obtained under local anesthesia without tonsillectomy. Since the introduction of human palatine tonsils as a new potential tissue source for MSCs in 2008, several studies have evaluated the characteristics of human palatine tonsil-derived MSCs (TMSCs) [2,3,4,5]. We previously found that the stem cells are located in the perivascular area of the palatine tonsil using a novel marker for endometrial MSCs, W5C5 [5], and their proliferation potential, which is the most important functional factor for stem cells, was significantly higher than that of bone marrow-derived MSCs (BMSCs) or adipose tissue-derived MSCs (ADSCs) [4]. Additionally, the palatine tonsil may be an alternative source for both autologous or allogenic MSC therapy, as donor age, long-term passage culture, and cryopreservation of the organ tissue do not significantly affect the characteristics of TMSCs [3].

In another previous study [4], we compared gene expression levels in TMSCs, ADSCs, and BMSCs to identify factors critical to the high proliferative properties of TMSCs. Fibroblast growth factor-5 (FGF5) expression was significantly higher in TMSCs than in ADSCs and BMSCs, and the regulatory mechanism of TMSC proliferation by FGF5 was determined. DNA microarray analysis showed that in addition to FGF5, tensin-3 (TNS3) expression was higher in TMSCs than in BMSCs and ADSCs (GEO accession number: GSE77272).

Tensin is a focal adhesion protein, of which TNS3 is one of the four tensin isoforms [6,7,8]. Focal adhesion, a structure in which various molecules are gathered around integrin, plays an important role in signal transduction and determining the mechanical linkage between the cell and extracellular matrix (ECM). Tensin binds competitively with other focal adhesion molecules (e.g., talin or kindlin) to the cytoplasmic tail of integrin beta-1 (ITGβ1) and is involved in cell adhesion or migration. However, the role of TNS3 in stem cells is not well-understood. In this study, we investigated the role of TNS3 in the proliferation and differentiation of TMSCs.

## 2. Materials and Methods

### 2.1. Culture of Palatine TMSCs, ADSCs, and BMSCs

The palatine tonsils were obtained from four different patients diagnosed with chronic tonsillitis who had undergone a tonsillectomy. To isolate the stem cells, the tonsils were washed extensively with equal volumes of phosphate-buffered saline (PBS), and the tissue was digested in 0.075% collagenase type I (Sigma-Aldrich, St. Louis, MO, USA) at 37 °C for 30 min. Enzyme activity was neutralized with alpha-modified Eagle’s medium (α-MEM) containing 10% fetal bovine serum (FBS), and the sample was centrifuged at 1200× *g* for 10 min. After centrifugation, the pellet was filtered through a 100-μm nylon mesh to remove cellular debris, and the filtrate was incubated overnight in control medium (α-MEM, 10% FBS, 100 U/mL of penicillin, and 100 μg/mL of streptomycin) at 37 °C under a 5% CO_2_ atmosphere. Following incubation, the plates were washed extensively with PBS to remove residual non-adherent cells, and the resulting cell populations were further maintained. All assays, including TNS3 blocking studies, were repeated three to four times in all four TMSCs. In this study, we utilized the cells that we verified the characteristics of mesenchymal stem cells by determining the proliferation, differentiation, and surface markers, as we previously reported [4,5].

ADSCs and BMSCs were isolated and characterized, as described in our previous studies [9,10]. The adipose tissues were obtained from abdominoplasties. To isolate the ADSCs, the adipose tissue samples were washed with PBS and digested in 0.075% collagenase type I at 37 °C for 30 min. Enzyme activity was neutralized with α-MEM containing 10% FBS. The samples were centrifuged at 1200× *g* for 10 min, and the pellet was incubated overnight in the control medium at 37 °C under 5% CO_2_. Following incubation, the tissue culture plates were washed to remove any residual non-adherent cells and then maintained in control medium at 37 °C under 5% CO_2_. Bone marrow samples were obtained from four volunteers. Mononuclear cells from the bone marrow were separated by centrifugation in a Ficoll–Hypaque gradient (density = 1.077 g/cm^3^; Sigma-Aldrich) and suspended in α-MEM containing 10% FBS, 100 U/mL of penicillin, and 100 μg/mL of streptomycin. The cultures were maintained at 37 °C in a humidified atmosphere containing 5% CO_2_. The adherent cell monolayer at 90% confluence was trypsinized (0.25% trypsin; Sigma-Aldrich), and the cells were resuspended in α-MEM containing 10% FBS and subcultured at a concentration of 2000 cells/cm^2^. Cells between the third and fourth passages were used in all further experiments.

The study protocol was reviewed and approved by the Pusan National University Hospital Institutional Review Board.

### 2.2. Long-Term Passage Culturing of Palatine TMSCs

Adherent primary TMSCs were expanded in culture, and colonies started to form after 5–6 days of isolation. The medium was replenished twice weekly. When cells reached 80–90% confluency, they were detached with a 0.25% trypsin/EDTA solution (Gibco, Grand Island, NY, USA). Population doubling and cell viability were measured. Next, the cells were seeded into culture flasks at a density of 1.5 × 10^3^ cells/cm^2^ with Dulbecco’s modified Eagle’s medium–low glucose containing 10% MSC-qualified FBS and incubated in a 37 °C incubator under 5% CO_2_. The cells were subcultured every 4–5 days to reach P28.

### 2.3. Quantitative Reverse Transcription-Polymerase Chain Reaction

Quantitative reverse transcription (RT)-polymerase chain reaction (PCR) was performed to determine the expression levels of TNS3, SOX2, Oct-4, Nanog, c-Myc, p16, p19, p21, CDC25, cyclin E, peroxisome proliferator-activated receptor-gamma (*PPARγ*), lipoprotein lipase (*LPL*), osteocalcin, alkaline phosphatase (*ALP*), collagen type II alpha-1 (*COL2A1*), and GAPDH as the internal control. Primer sequences are shown in Table 1 and were determined using established GenBank sequences. Real-time quantification was based on the LightCycler assay using a fluorogenic SYBR Green I PCR mixture with a LightCycler instrument (Roche, Mannheim, Germany). LightCycler version 3.3 software was used to analyze the PCR kinetics and quantitative data. All experiments were conducted three times, and negative and positive controls were included in all experiments. 

### 2.4. Western Blot Analysis

The cells were washed twice in cold PBS and lysed in RIPA buffer (Sigma-Aldrich). Proteins were loaded into a 10% sodium dodecyl sulfate-polyacrylamide gel, electrotransferred to nitrocellulose membranes (Hybond-ECL; Amersham Pharmacia Biotech, Piscataway, NJ, USA), and probed with monoclonal antibodies. Immunoreactive bands were detected using anti-mouse and anti-rabbit peroxidase-conjugated secondary antibodies (Amersham Pharmacia Biotech) and visualized by enhanced chemiluminescence (ECL detection kit; Amersham Pharmacia Biotech).

### 2.5. Transfection of siRNA

Small interfering RNA (siRNA) oligonucleotide duplexes (ON-TARGETplus SMARTpool; Dharmacon, Thermo Scientific, Epsom, UK) targeting TNS3 and ITGβ1 mRNAs, and a nontargeted oligonucleotide duplex (ON-TARGETplus siCONTROL; Dharmacon) as a negative control, were used in the transfection experiments with DharmaFECT Transfection Reagent. 

### 2.6. Cell Proliferation 

Cell proliferation was evaluated by the 3-(4,5-dimethylthiazol-2-yl)-2,5-diphenyltetrazolium bromide (MTT) assay and cell cycle analysis. For the MTT assay, TMSCs were plated at a density of 1 × 10^4^ cells/well in 24-well plates and evaluated after 1, 2, 3, and 4 days of culture. After washing the cells, the culture medium containing 0.5 mg/mL of MTT was added to each well. The cells were incubated for 2 h at 37 °C, after which the supernatant was removed, and formazan crystals that had formed in viable cells were solubilized with 200 μL of dimethyl sulfoxide. Next, a 100-μL aliquot of each sample was transferred into 96-well plates, and the absorbance of each well was measured at 560 nm using an ELISA reader (XFLUOR4 software, version 4.51, Thomas Scientific, Swedesboro, NJ, USA). This experiment was repeated four times. Cell cycle analysis was performed using 6-well plates seeded with 5 × 10^4^ cells/well. These cells were treated with recombinant human FGF5 for 48 h at 37 °C and harvested using 0.05% trypsin solution. Thereafter, they were centrifuged at 10,000× *g* for 15 min, and the pellets were washed twice in Hank’s Balanced Salt Solution buffer and fixed with 70% ethanol at −20 °C overnight. On the following day, ethanol was removed, and the cells were resuspended in 500 mL of PBS containing 1 mg/mL of propidium iodide and 100 μg of RNase/mL for 20 min, followed by analysis with a FACS Calibur (BD Biosciences, San Jose, CA, USA).

### 2.7. Cell Migration Assay

TMSC migration was analyzed using Transwell chambers with an 8-μm pore size. The cells (4 × 10^5^) were plated into the upper chamber, while the lower chamber was filled with media containing interferon-γ and tumor necrosis factor α. The Transwell chambers were incubated for 24 h to allow cell migration toward inflammatory cytokines. The cells were fixed with 10% formaldehyde, and cells on the upper side of the chamber, which had not migrated through the pore, were removed with cotton swabs. The remaining migrated cells were stained with DAPI. After washing, stained cells were counted under a fluorescence microscope (Leica Microsystems, Wetzlar, Germany).

### 2.8. Senescence-Associated Beta-Galactosidase Staining

Senescence-associated beta-galactosidase staining was conducted as previously described [11]. Briefly, TMSCs were seeded into 6-well plates and incubated to reach 70–80% confluency. The cells were washed and fixed with glutaraldehyde (0.5%, pH 7.2) for 5 min at room temperature followed by washing with PBS containing MgCl_2_. The cells were stained with X-gal solution containing 1 mg/mL X-gal, 0.12 mM K_3_Fe(CN)_6_, and 1 mM MgCl_2_ overnight at 37 °C. After washing, images were captured.

### 2.9. Colony Formation Assay

A colony formation assay was performed by plating the cells in 6-well plates (100 cells/well). After 2 weeks, the cells were washed twice, followed by fixation with ethanol 70%, 15 min), and staining with crystal violet (0.5%) at room temperature. After washing, images were captured.

### 2.10. Multi-Lineage Differentiation 

The potential of TMSCs to differentiate into the adipogenic, osteogenic, and chondrogenic lineages was analyzed. Adipogenic differentiation was induced by culturing TMSCs in adipogenic medium (α-MEM supplemented with 10% FBS, 1 µM dexamethasone (Sigma-Aldrich), 100 μg/mL 3-isobutyl-l-methylxanthine, 5 μg/mL insulin, and 60 μM indomethacin) for 3 weeks, and assessed by Oil Red O (Sigma-Aldrich) staining as an indicator of intracellular lipid accumulation. Before staining, the cells were fixed in 70% ethanol for 15 min at room temperature. The cells were then incubated in 2% Oil Red O reagent for 1 h at room temperature. To visualize lipid droplets, the excess stain was removed by washing with 70% ethanol and then distilled water. Osteogenic differentiation was induced by culturing the TMSCs for 3 weeks in osteogenic medium (α-MEM supplemented with 10% FBS, 0.1 mM dexamethasone, 10 μM β-glycerophosphate, and 50 μg/mL ascorbic acid), with an examination of ECM calcification conducted by Alizarin Red S (Sigma-Aldrich) staining. Briefly, after fixation with 70% ethanol and washing with distilled water, the cells were incubated in 2% Alizarin Red solution for 15 min at room temperature and then washed numerous times with distilled water. Chondrogenic differentiation was induced using the micromass culture technique. Briefly, 10 μL of a concentrated MSC suspension (3 × 10^5^ cells/mL) was plated into the center of each well and allowed to attach at 37 °C for 2 h. Chondrogenic medium (α-MEM supplemented with 1% FBS, 0.1 mM dexamethasone, 50 μg/mL ascorbic acid, insulin–transferrin–selenium (ITS+1; Sigma-Aldrich), and 10 ng/mL transforming growth factor beta-1 (Sigma-Aldrich)) was gently overlaid so as to not detach the cell nodules, and the culture was maintained in the medium for 4 weeks before analysis. Chondrogenesis was confirmed by immunohistochemistry for collagen type-II staining. The sections were blocked with 10% horse serum, incubated with purified anti-mouse COL2A1 antibody (BD Biosciences) for 1 h, and then washed with PBS (pH 7.4). Antibody-bound cells were detected with a peroxidase substrate kit (Vectastain ABC kit; Vector Laboratories, Burlingame, CA, USA). The sections were then washed, counterstained with hematoxylin, and examined by light microscopy.

### 2.11. Flow Cytometry 

Flow cytometric analysis was used to measure the total and active cell-surface ITGβ1 levels of the TMSCs. At least 50,000 cells (in 100 μL of PBS containing 0.5% bovine serum albumin and 2 mM EDTA) were incubated with phycoerythrin-conjugated monoclonal antibodies against human total and active ITGβ1 (BD Biosciences). The labeled cells were analyzed by flow cytometry using a FACSCaliber flow cytometer equipped with CellQuest Pro software (BD Biosciences).

### 2.12. Immunocytochemical Staining

Cells were fixed with 4% paraformaldehyde for 10 min, permeabilized with 0.5% Tween 20 for 10 min, and blocked in 1% bovine serum albumin for 1 h at room temperature. The fixed cells were stained with purified rabbit anti-human ITGβ1 at 4 °C for 2 h and then incubated with phycoerythrin-conjugated goat anti-rabbit IgG (e-Bioscience, San Diego, CA, USA) and fluorescein isothiocyanate-conjugated goat anti-mouse IgG (e-Bioscience) for 1 h at room temperature. The cells were mounted with 4′,6-diamidino-2-phenylindole containing the VECTASHIELD (Vector Laboratories) mounting medium and examined using a confocal laser-scanning microscope.

### 2.13. Statistical Analysis

The data are expressed as the mean ± standard deviation in all experiments. One-way analysis of variance (SPSS version 19.0 software; SPSS, Inc., Chicago, IL, USA), followed by Scheffé’s test, was conducted to detect significant differences between groups. A *p* value of <0.05 was considered statistically significant.

## 3. Results

### 3.1. Tensin-3 Regulates the Proliferation of Palatine TMSCs 

**Figure 1:**
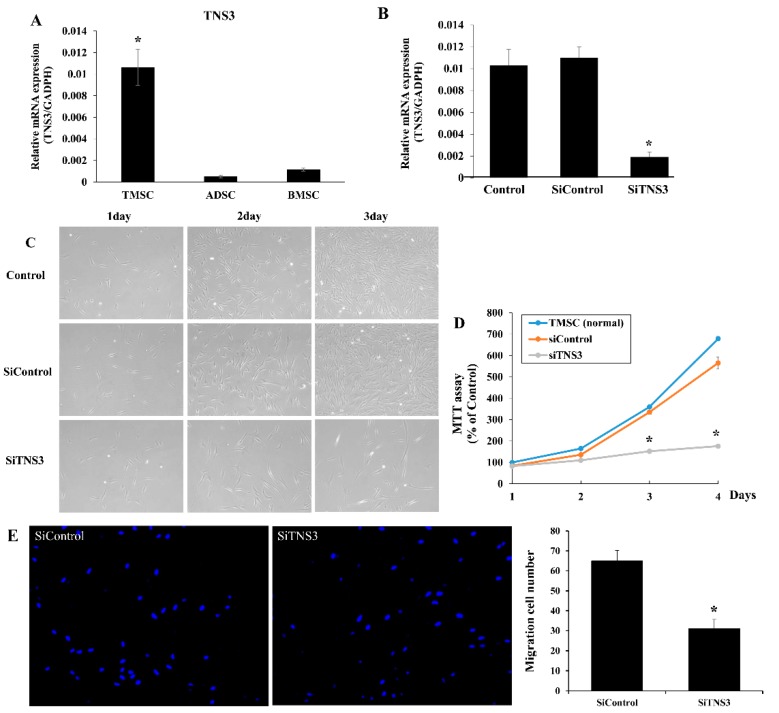
Tensin-3 (TNS3) regulates the proliferation of tonsil mesenchymal stem cells (TMSC). (**A**) TNS3 showed increased expression levels in TMSCs compared to in adipose tissue-derived MSCs (ADSCs) and bone marrow-derived MSCs (BMSC). (**B**) siRNA TNS3 (siTNS3) transfection of TMSCs significantly reduced TNS3 expression in RT-PCR. (**C**) Photomicrographs after siTNS3 transfection showed reduced cell numbers, but no morphologic changes were observed in transfected cells compared to controls. (**D**) 3-(4,5-dimethylthiazol-2-yl)-2,5-diphenyltetrazolium bromide (MTT) assay results. Silencing of TNS3 led to a decrease in cell growth rates. The difference compared to the control was significant on days 3 and 4. (**E**) Migration cell numbers were also significantly reduced after transfection with siTNS3. *, *p* < 0.05 compared to the control. *n* = 4. Columns and error bars represent the mean ± standard deviation.

To confirm that TNS3 is more highly expressed in TMSCs than in the other cells, RT-qPCR was conducted to detect TNS3 mRNA in TMSCs for comparison with that in ADSCs and BMSCs from four different donors. TNS3 expression in TMSCs was significantly higher than in ADSC and BMSC cultures (*p* < 0.05) (Figure 1A). 

Next, to determine the effect of TNS3 on TMSC proliferation, changes in cell growth after transfection with siRNA TNS3 (siTNS3) were examined. RT-qPCR revealed that the expression of TNS3 was significantly downregulated in siTNS3-transfected TMSCs (Figure 1B). Phase-contrast photomicrography revealed no change in cell morphology after siTNS3 treatment. The MTT assay was performed to quantify the proliferation rate. Transfection with siTNS3 led to a reduction in the cell growth rate, with significant differences observed on days 3 and 4 (control 340 ± 31.0% vs. siTNS3 110 ± 3.3%, *p* < 0.05 on day 3; control 550 ± 40% vs. siTNS3 115 ± 3.3%, *p* < 0.05 on day 4). Moreover, the migration of TMSCs towards inflammatory cytokines was significantly suppressed by siTNS3 transfection (Figure 1C–E).

**Figure 2:**
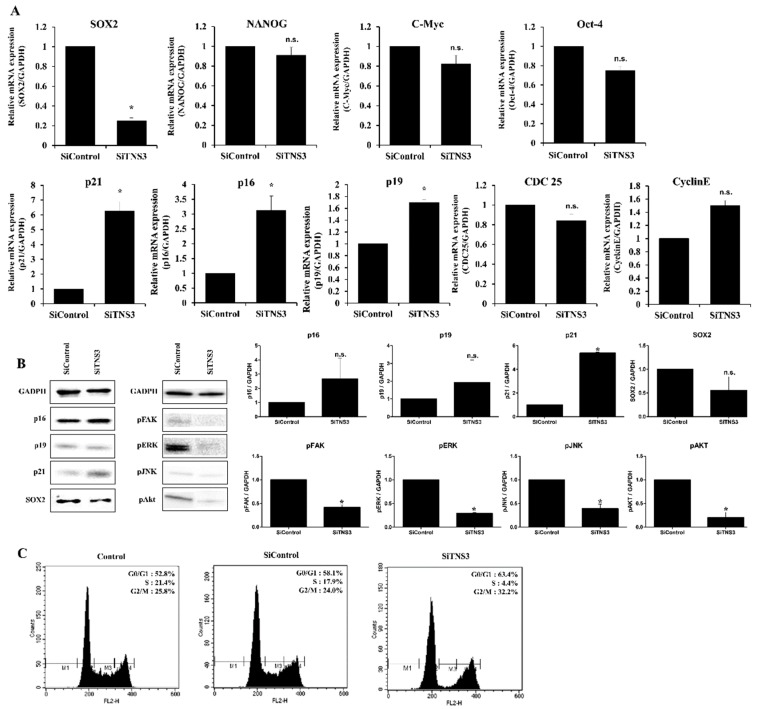
TNS3 is involved in cell cycle regulation and the pluripotency of TMSCs. After siTNS3 treatment, p16 was increased, and SOX2 was inversely decreased in both RT-qPCR (**A**) and Western blotting assays (**B**). Extracellular signal-regulated kinase (ERK), protein kinase B (AKT), and c-Jun N-terminal kinase (JNK) levels were all decreased after siTNS3 treatment, with that of ERK being lower (**B**). (**C**) For cell cycle analyses, 6-well plates were seeded with 5 × 10^4^ cells/well and analyzed with the FACS Calibur system. The proportion of active phase cells (S + G2/M) was reduced, and that in the G1/G0 phase was increased. *, *p* < 0.05 compared to the control. n.s., not significant compared to the control. *n* = 3. Columns and error bars represent the mean ± standard deviation.

p16 and p21 are associated with cell cycle regulation and are frequently measured as markers of cellular senescence. After siTNS3 treatment, p16 and p21 (associated with cell cycle regulation) were increased at both the mRNA and protein levels. Among well-known pluripotency markers in stem cells, SOX2 expression was downregulated by TNS3 inhibition, whereas the expression of Nanog, c-Myc, and Oct-4 was not affected. Additionally, phosphorylation of focal adhesion kinase (FAK), extracellular signal-regulated kinase (ERK), protein kinase B (PKB, also known as Akt), and c-Jun N-terminal kinase (JNK) was decreased after siTNS3 treatment (Figure 2A,B). Cell cycle analysis showed that 58.1% of TMSCs were in the G1/G0 phase, 17.9% were in the S phase, and 24.0% were in the G1/M phase. Following siTNS3 treatment, 63.4% of the cells were in the G1/G0 phase, 4.4% were in the S phase, and 32.2% were in the G2/M phase, indicating that the number of cells in the S phase was reduced and that in the G1/G0 phase was increased (Figure 2C). These results suggest that TNS3 regulates the self-renewal of TMSCs mainly by suppressing SOX2 and signaling pathways critical for cell survival and proliferation. 

### 3.2. Replicatively-Senescent TMSCs Exhibit the Senescence Phenotype and Decreased TNS3 Expression

**Figure 3:**
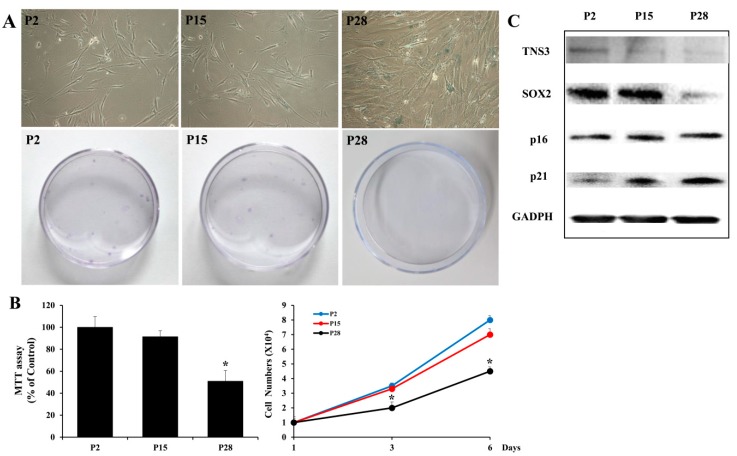
TNS3 is involved in regulating TMSC senescence. (**A**) Beta-galactosidase staining and colony-forming unit assay were performed in early, intermediate, and late passages of TMSCs. (**B**) MTT assay was conducted to evaluate the proliferation of TMSCs at different passages. (**C**) Protein expression of TNS3, SOX2, p16, and p21 was detected in TMSCs at different passages by Western blotting. *, *p* < 0.05 compared to the control. *n* = 3. Columns and error bars represent the mean ± standard deviation.

Given that critical factors involved in MSC proliferation are related to the senescence of MSCs [12,13,14,15], we next investigated whether TNS3 is involved in the senescence phenotype of TMSCs by analyzing TMSCs from early (P2), intermediate (P15), and late (P28) passages after relevant consecutive subcultures. Typical phenotypes reported in replicative senescence of MSCs, including a flattened and enlarged morphology, were observed in late passage TMSCs. Colony formation units for late passage TMSCs were lower than those for early or intermediate passage TMSCs. Moreover, late passage TMSCs exhibited higher activity of senescence-associated β-galactosidase compared to the activity in early and intermediate passage MSCs (Figure 3A). Similarly, late passage MSCs showed a decreased proliferation rate, whereas early and intermediate passage cells maintained their ability to proliferate (Figure 3B). Interestingly, TNS3 expression was downregulated in late passage TMSCs, along with decreased SOX2 expression and increased p21 expression, similar to our observations in TNS3 inhibition analysis (Figure 3C). These results indicate that the replicative senescence of TMSCs is at least partially affected by the maintenance of TNS3 expression.

### 3.3. Tensin-3 Plays a Role in the Differentiation of Palatine TMSCs

**Figure 4:**
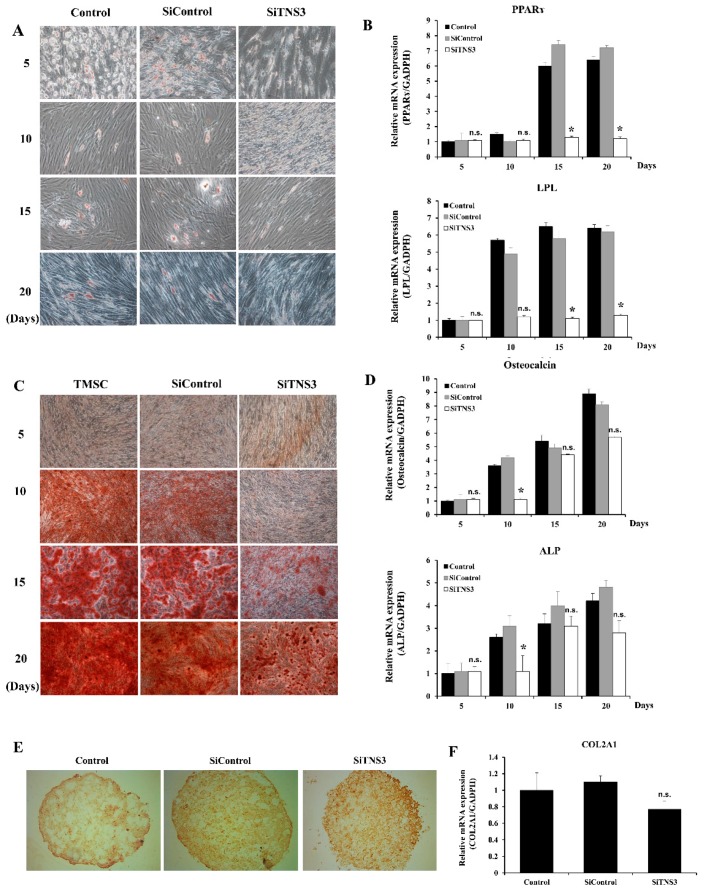
TNS3 plays an important role in the differentiation of TMSCs. (**A**,**B**) Adipogenic differentiation. Transfection with siTNS3 reduced the number of cells containing lipid droplets during the whole study period in the Oil Red O staining assay (**A**). RT-qPCR revealed that the siTNS3-transfected TMSCs had reduced expression of adipogenic transcripts (PPARγ and LPL) after induction for 15 and 20 days (**B**). (**C**,**D**) Osteogenic differentiation. Enhanced Alizarin Red S staining was observed from day 10 in the control group, whereas weak staining was observed even on days 15 and 20 in siTNS3-transfected TMSCs, where the level of calcium deposition was much lower than that in the control (**C**). Treatment with siTNS3 reduced the expression of osteocalcin and ALP in the control on day 10 (**D**). (**E**,**F**) Chondrogenic differentiation. Collagen type II immunohistochemical staining was slightly reduced in siTNS3-transfected TMSCs compared to in the control group (**E**). *COL2A1* expression was lower in siTNS3-transfected TMSCs than in the control group (**F**). *, *p* < 0.05 compared to the control. n.s., not significant compared to the control. *n* = 3. Columns and error bars represent the mean ± standard deviation.

To investigate the role of TNS3 in TMSC differentiation into adipocytes, the cells were cultured in adipogenic differentiation medium and then stained with Oil Red O to observe lipid accumulation. After induction for 5, 10, 15, and 20 days, typical adipocytes and lipid droplets were observed in the adipogenesis-induced group and siControl-treated cells. In contrast, the treatment of TMSCs with siTNS3 reduced the number of cells containing lipid droplets throughout the study period (Figure 4A). RT-qPCR revealed that siTNS3-transfected TMSCs exhibited reduced expression of adipogenic transcripts (PPARγ and LPL) at days 15 and 20 after differentiation induction (*p* < 0.05) (Figure 4B). For osteogenic differentiation analysis, TMSCs cultured in osteogenic differentiation medium were stained with Alizarin Red S to evaluate calcium deposition. Enhanced Alizarin Red S staining was observed from day 10 in the differentiation-induced group, whereas weak staining was observed even on days 15 and 20 in siTNS3-transfected TMSCs, in which minimal calcium deposition was detected (Figure 4C). Treatment with siTNS3 led to reduced expression of osteogenic differentiation markers (osteocalcin and ALP) (*p* < 0.05) (Figure 4D). The role of TNS3 in the chondrogenic differentiation of TMSCs was also evaluated by collagen type II immunohistochemical staining. The results showed that TNS3 inhibition slightly reduced the intensity of collagen type II staining (Figure 4E). RT-qPCR revealed a lower expression of *COL2A1* in siTNS3-transfected TMSCs (Figure 4F). These results suggest that TNS3 is involved in the downregulation of adipogenesis, osteogenesis, and chondrogenesis of TMSCs, as determined by specific staining and transcript analyses.

### 3.4. Tensin-3 Controls Integrin Beta-1 Activity

**Figure 5:**
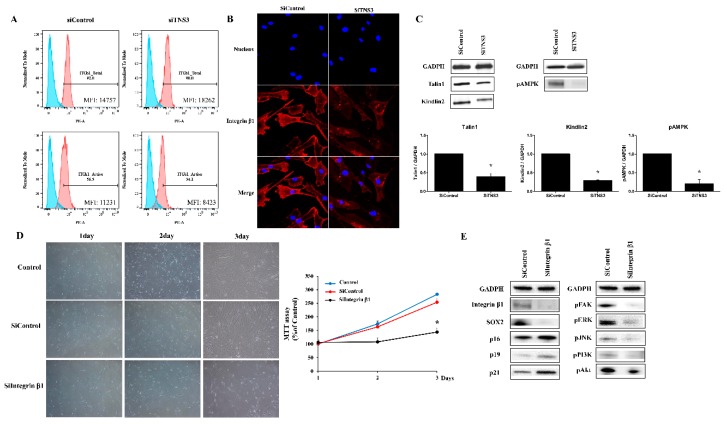
TNS3 controls the activity of ITGβ1. (**A**) Total and active ITGβ1 levels were 80.9% and 80.5%, respectively, in siControl-treated TMSCs, and were significantly reduced to 51.2% and 49.2%, respectively, following siTNS3 treatment by flow cytometry. (**B**) After siTNS3 treatment, both nucleus (blue) and ITGβ1 (red) decreased. Confocal microscopy (×400). (**C**) When TNS3 was inhibited, talin, kindlin were unchanged, and AMPK was reduced in Western blot analysis. (**D**) Photomicrographs acquired after siTNS3 transfection showed reduced cell numbers, but no morphologic changes in transfected cells compared to the control (left panel). In the MTT assay, the siITGβ1-treated group showed lower proliferation rates compared to the control during the whole observation period; these differences were significant on day 3. (**E**) In Western blot analysis, the level of SOX2 was decreased, whereas those of p16, p19, p21 were increased after siITGβ1 treatment. When ITGβ1 was inhibited, the pFAK, pERK,p JNK,pPI3K,pAkt levels were all decreased. * *p* < 0.05 compared to the control. *n* = 4. Columns and error bars represent the mean ± standard deviation.

To investigate the effect of TNS3 on ITGβ1, integrin activity was measured after inhibition of TNS3. The proportions of cells expressing total and active ITGβ1 levels were detected in siTNS3-treated and siControl-treated TMSCs, respectively. Active ITGβ1-positive cells were decreased after siTNS3 treatment, whereas total form expression was not altered (Figure 5A). Consistently, the intensity of active ITGβ1 staining was reduced by siTNS3 treatment. We next investigated the distribution of active ITGβ1, which is related to the adhesion pattern. Interestingly, active integrins were located near the center of the cells when TNS3 expression was inhibited, whereas they were densely distributed along the edges of cells in the siControl group, indicating that TNS3 is involved in regulating focal and fibrillar adhesion of cells via integrin distribution (Figure 5B). Moreover, the expression of talin1 and kindlin2, known as integrin regulators, was downregulated after TNS3 suppression. Additionally, phosphorylation of AMPK, a key factor in cellular metabolism regulation, was decreased after siTNS3 treatment (Figure 5C).

To investigate the correlation between signaling regulated by TNS3 and integrin, ITGβ1 expression was suppressed by transfection of siRNA for ITGβ1, and defined targets in TNS3 inhibition analysis were analyzed in parallel. Transfection with siITGβ1 led to a significant reduction in the cell growth rates in both the imaging and MTT assays (siControl 254.0 ± 15.8% and siITGβ1 144.0 ± 4.3%, *p* < 0.05 on day 3) (Figure 5D). To further evaluate the relationship between ITGβ1 and TNS3 in TMSC biology, changes in the levels of p16, p19, p21, and SOX2 were detected by Western blot analysis after transfection of the cells with siITGβ1. As observed following knockout of TNS3, the silencing of ITGβ1 increased the levels of p16 and p21 and decreased the level of SOX2. Additionally, the phosphorylation levels of FAK, ERK, phosphoinositide 3-kinase (PI3K), Akt, and JNK were decreased (Figure 5E). Taken together, these results indicate that TNS3 controls the biological activity of TMSCs by regulating the activity of ITGβ1.

## 4. Discussion

The proliferative capacity and multilineage differentiation potential of stem cells are critical for their clinical application. In a previous study [4], we showed that TMSCs have a higher proliferative potential than ADSCs and BMSCs. In this present study, we found that TMSCs had competitive differentiation potential and proliferative capacity, characteristics that are closely related to TNS3.

Tensin is a cytoplasmic phosphoprotein located at focal adhesion sites. This multidomain protein can interact with several molecules and is divided into four types depending on the structure of its N-terminus [8]. One of the four tensin isoforms, TNS3, has three types of domains. The N-terminus has actin-binding domains that interact with actin filaments. The opposite C-terminus has an Src homology 2 domain and phosphotyrosine-binding domain. The Src homology 2 domain interacts with tyrosine-phosphorylated proteins, including FAK, PI3K, and p130Cas. The phosphotyrosine-binding domain binds to the tail of the integrin molecule [16]. Tensin functions as a bridge between the ECM and cell because its structural features allow it to bind to both focal adhesion sites and actin at both ends. These structural properties, similar to talin, allow TNS3 to connect actin and integrin to transfer mechanical forces from both sides. The integrin-tensin complex is deeply involved in cell adhesion and migration [6]. It also acts as a platform for various tyrosine-phosphorylated signaling complexes and serves as a biochemical signaling hub between the ECM and cells through focal adhesion [8,17]. TNS3 is known to be distributed in many tissues, including the heart, skeletal muscle, kidney, lung, small intestine, liver, colon, prostate, ovary, and testis [18,19]. Recently, it was reported that TNS3 contributes to oncogenesis in human cancer cell lines [20,21]. However, the mechanisms behind these various roles of tensin remain unclear, and the function of TNS3 in stem cells has not been reported.

In this study investigating the role of TNS3 in TMSC functions, basal expression of TNS3 was found to be significantly more abundant in TMSCs than in ADSCs or BMSCs. Additionally, in siTNS3-transfected TMSCs, cell proliferation was significantly decreased, along with an increase in the G0/G1 phase and a decrease in the S phase, indicating cell cycle arrest. TNS3 plays an important role in the differentiation and proliferation of TMSCs, as adipogenic, osteogenic, and chondrogenic differentiation was reduced when TNS3 was downregulated in the cells. In particular, adipogenic differentiation was significantly reduced when TMSCs were treated with siTNS3.

Integrins, transmembrane receptors composed of 18 alpha-subunits and 8 beta-subunits, are the central molecule in focal adhesion [22]. Integrin modulates cell growth, division, survival, and apoptosis through various receptor tyrosine kinases, such as FAK, ERK, mitogen-associated protein kinase, and PI3K [23,24,25,26]. The role of integrin in stem cells has been examined previously [27,28,29,30,31], and it is well-known that its modulation of the intra- and extracellular signaling pathways influences the migration, proliferation, and differentiation of MSCs. In focal adhesion, the cytodomain of ITGβ1 binds focal adhesion proteins connected to actin. Tensin binds to the tail of ITGβ1 in competition with these focal adhesion proteins [8,32]. In a study by Georgiadou et al. [33], the activity of ITGβ1 was increased when the talin attached to the integrin molecule was detached and attached to TNS3; in contrast, TNS3 silencing induced a significant reduction in integrin activity in fibroblasts. These studies suggested that TNS3 regulates integrin activity in fibrillogenesis. 

In the present study, ITGβ1 activity was significantly reduced after siTNS3 treatment. When ITGβ1 was blocked, its downstream signaling molecules (ERK, JNK, and PI3K) were decreased, which was similar to the results observed in TNS3 silencing. Moreover, the alterations in p16, p21, and SOX2 expression were consistent with the change observed in TNS3 inhibition. The findings indicate that TNS3 regulates the activity of ITGβ1 and influences the behavior of TMSCs.

However, this study has several limitations. First, we examined whether TNS3 is significantly more expressed in TMSCs than in BMSCs or ADSCs, and thus TNS3 was the only tensin isoform investigated. The other tensin isoforms with structures similar to that of TNS3 may also have important functions in TMSCs. Particularly, because TNS4 is also known to play a complementary role to TNS3 [34,35,36], further studies of other tensin isoforms, including TNS4, are necessary. Second, we did not determine why adipogenic differentiation was more significantly reduced than osteogenic or chondrogenic differentiation when TNS3 was blocked. Third, we did not examine the details of the relationship between talin and kindlin, which controls integrin, and TNS3, although we showed that their expression was decreased. Finally, the limitation of our findings is the lack of direct relevance to clinical application, as well as the evidence for pivotal function as cell therapeutics. Because TNS3 not only regulates the major characteristics of stem cells, including proliferation and differentiation but is also involved in the migration of cells by regulating adhesion patterns, one can envision that TNS3 might play a pivotal role in the therapeutic efficacy of TMSCs after transplantation through the regulation of cell migration. Further studies investigating the role of TNS3 in therapeutic application and exploring the strategy to regulate TNS3 for improved efficacy are required to prove the clinical relevance of TNS3 in TMSC application. The therapeutic application of MSCs, including angiogenesis, regeneration of bone or muscle, wound healing, and immunomodulation, should be evaluated using in vivo models after inhibition or over-expression of TNS3.

In conclusion, our results show that TNS3 increased the proliferative capacity and differentiation potential of TMSCs by regulating ITGβ1 activity.

## Figures and Tables

**Table 1:** Primer sequence used in real-time polymerase chain reaction (PCR).

Gene	Primer Sequences
TNS3	Forward 5′-GGACGCATAGGAGTGGTGAT-3′ Reverse 5′-GGGAGAGGCATTCATTTTCA-3′
SOX2	Forward 5′-GGGAAATGGGAGGGGTGCAAAAGAGG-3′ Reverse 5′-TTGCGTGAGTGTGGATGGGATTGGTG-3′
NANOG	Forward 5′-AGTCCCAAAGGCAAACAACCCACTTC-3′ Reverse 5′-TGCTGGAGGCTGAGGTATTTCTGTCTC-3′
Oct-4	Forward 5′-GACAGGGGGAGGGGAGGAGCTAGG-3′ Reverse 5′-CTTCCCTCCAACCAGTTGCCCCAAAC-3′
C-myc	Forward 5′-AAAGGCCCCCAAGGTAGTTA-3′ Reverse 5′-GCACAAGAGTTCCGTAGCTG-3′
p16	Forward 5′-GGGGAGAGTAGATAGCGGGC-3′ Reverse 5′-AACCAATCAACCGAAAATTCCATA-3′
p19	Forward 5′-CTCTGCTCCCTGATAGCCCT-3′ Reverse 5′-TGCGAAGGATTTTGAAGCGG-3′
p21	Forward 5′-GTCACTGTCTTGTACCCTTGTG-3′ Reverse 5′-CGGCGTTTGGAGTGGTAGAAA-3′
CDC25	Forward 5′-CTTCCTTTACCGTCTGTC-3′ Reverse 5′-AAACCATTCGGAGTGCTA-3′
Cyclin E	Forward 5′-GGATGTTGACTGCCTTAG-3′ Reverse 5′-CACCACTGATACCCTGAAA-3′
GAPDH	Forward 5′-CCTACACCACCAACTGCTTA-3′ Reverse 5′-GGCCATCCACAGTCTTCTGAG-3′
